# Effects of Tomato Water Stress on the Life-History and Population Parameters of the Predatory Thrips *Scolothrips longicornis*

**DOI:** 10.3390/plants15213250

**Published:** 2026-10-22

**Authors:** Hajar Pakyari, Rostislav Zemek

**Affiliations:** 1 Department of Plant Protection, Tak. C., Islamic Azad University, Takestan P.O. Box 34819-49479, Iran; 2 Institute of Entomology, Biology Centre CAS, 370 05 Ceske Budejovice, Czech Republic; rosta@entu.cas.cz

**Keywords:** drought, bottom-up effects, multi-trophic interactions, spider mites, *Tetranychus urticae*, *Solanum lycopersicum*, age-stage, two-sex life table, biological control, population projection

## Abstract

Increasing drought occurrence under climate change is expected to alter ecological interactions in agroecosystems, with important implications for biological control. Drought can alter plant–herbivore interactions and thereby affect the performance of natural enemies through bottom-up effects. However, the demographic consequences of drought-induced changes in host plants for predatory thrips remain poorly understood. We investigated the effects of three soil moisture treatments of tomato plants on the life-history and demographic performance of the predatory thrips *Scolothrips longicornis* Priesner feeding on *Tetranychus urticae* Koch eggs under constant abiotic conditions (25 ± 1 °C, 65 ± 5% RH, and a 16L:8D photoperiod) in a controlled-environment growth chamber. The plants were maintained under 100% field capacity (100% FC), moderate water stress (70% FC), or severe water stress (50% FC). The soil moisture treatment significantly affected the duration of the egg stage but not that of other immature stages and did not have an effect on pre-adult survival. In contrast, severe water stress significantly reduced female and male longevity. Reproductive performance showed a nonlinear response to water availability. Females under 70% FC had significantly higher fecundity and a longer oviposition period than females under 100% FC or severe water stress. The net reproductive rate was the highest under 70% FC and differed significantly from both 100% FC and 50% FC. Similarly, the intrinsic rate of increase, finite rate of increase, and mean generation time were significantly affected by treatment. Overall, moderate water stress maintained or enhanced several fitness-related traits of *S. longicornis*, whereas severe water stress reduced adult longevity and reproductive performance. These findings demonstrate that plant-mediated effects of drought on predatory thrips are nonlinear and highlight the importance of plant water status in determining the demographic potential of natural enemies and their effectiveness in biological control programs for water-limited tomato production systems.

## 1. Introduction

Drought and increasing water scarcity are among the major environmental challenges facing agricultural production under a changing climate. Changes in precipitation regimes and increasing frequency of water-deficit episodes can alter plant growth, physiology, resource allocation, and defensive traits, with consequences that extend beyond individual plants to interactions among plants, herbivores, and their natural enemies. Consequently, drought should not be considered solely as an abiotic constraint on crop productivity, but also as an ecological driver capable of restructuring multitrophic interactions in agroecosystems [1,2,3,4]. The consequences of water limitation for arthropod communities can be particularly complex because changes in plant quality and abundance may simultaneously influence herbivore performance and the abundance, survival, and effectiveness of higher trophic levels [2,5].

Water availability can modify plant–herbivore interactions through changes in primary metabolism, tissue water content, nutrient concentration, defensive chemistry, and plant volatile emissions. Depending on the intensity, duration, and timing of water limitation, these changes can either increase or decrease herbivore performance. Recent syntheses indicate that water deficit may enhance some constitutive resistance and escape traits while compromising plant tolerance and certain forms of indirect defense, including volatile-mediated attraction of natural enemies [2]. Experimental studies have likewise demonstrated that water stress can alter herbivore development, reproduction, and population dynamics, emphasizing that responses to drought are highly dependent on the particular plant–herbivore combination and the severity of water limitation [1,3,6].

These variable responses have traditionally been interpreted through several complementary hypotheses concerning plant–herbivore interactions. The Plant-Stress Hypothesis predicts that physiological stress can increase plant suitability for some herbivores through changes in plant nutritional quality, whereas the Plant-Vigor Hypothesis predicts that herbivores may perform better on vigorous, rapidly growing plants. The Pulsed-Stress Hypothesis further proposes that the temporal pattern and severity of water stress can determine whether plant stress benefits or constrains herbivore performance [1,7,8]. Recent research indicates that no single hypothesis adequately explains all plant–herbivore combinations and that outcomes depend on plant species and genotype, herbivore feeding guild, stress intensity, and the interactions occurring across trophic levels [2,3].

Importantly, drought-induced changes in plants can propagate to higher trophic levels through bottom-up effects. Changes in plant nutritional quality, morphology, and defensive chemistry can alter herbivore abundance and quality and, consequently, affect predators and parasitoids that depend on those herbivores. Field and experimental studies have shown that water limitation can reduce natural-enemy abundance or alter their interactions with herbivores, although the direction and magnitude of these effects are strongly system-dependent [2,5]. For example, drought-stressed plants have been shown to exhibit altered volatile-mediated indirect defense, potentially reducing attraction of natural enemies, while other systems have demonstrated increased herbivore performance under moderate water limitation [3,9,10]. These findings emphasize the importance of investigating specific plant–herbivore–natural enemy combinations rather than assuming a uniform response of biological control agents to drought.

Despite increasing evidence that abiotic stress can modify the performance of biological control agents, considerably less is known about the effects of plant water limitation on predatory thrips. This knowledge gap is important because the response of a predator feeding on herbivores developing on stressed plants represents a multitrophic process rather than a simple direct response of the predator to drought. Water limitation may alter host plant physiology and, consequently, the abundance, developmental performance, nutritional quality, or accessibility of prey. These changes can then be transmitted to predatory thrips, potentially modifying their development, survival, reproduction, and population growth. Thus, drought effects on the predator may emerge through a combination of plant-mediated bottom-up effects and changes in the predator–prey interaction [11].

Tomato (*Solanum lycopersicum* L.) provides an important model crop for investigating these interactions because it is widely cultivated under protected and water-limited production systems and supports numerous economically important arthropod pests. Among these, the two-spotted spider mite, *Tetranychus urticae* Koch (Acari: Tetranychidae), is a major pest that can develop rapidly on tomato and cause substantial damage by feeding on leaf tissues. Previous work has demonstrated that drought can alter the suitability of tomato for *T. urticae*. In particular, drought-stressed tomato plants increased the performance of tomato-adapted *T. urticae* populations, accompanied by changes in plant nutritional characteristics and defense-related traits [12]. More recent research on the effect of water stress on plant–herbivore interactions further demonstrates that changes in plant water status can substantially alter herbivore performance and plant defense, reinforcing the importance of water availability in determining the dynamics of tomato-associated arthropod communities [2,13].

The consequences of these plant-mediated changes are particularly important for biological control because *T. urticae* is attacked by many predatory arthropods. Among them, predatory thrips of the genus *Scolothrips* are specialized acarophagous predators and are considered promising biological control agents of tetranychid mites [14,15]. *Scolothrips longicornis* Priesner (Thysanoptera: Thripidae) develops successfully on *T. urticae*, exhibits a positive intrinsic rate of population increase, and can consume substantial numbers of *T. urticae* eggs during its immature and adult stages [16], confirming it has high potential for biological control of spider mites. Life-table analysis further demonstrated strong effects of prey species [17], temperature [14,15], light wavelength [18], short-term exposure to high temperatures [19], exposure to pesticide [20], and group rearing [21] on *S. longicornis* performance.

The direction of drought effects may be nonlinear. Moderate water stress can induce physiological changes in plants without necessarily causing severe deterioration of plant quality, potentially creating conditions that favor herbivore development or alter prey quality in ways that benefit their natural enemies. In contrast, severe water stress may impair plant growth and resource availability and ultimately reduce the quality or persistence of the herbivore resource and its natural enemies. Recent experimental studies provide evidence for such context-dependent responses. For example, moderate water stress increased the performance of a jujube lace bug, *Monosteira alticarinata* Ghauri (Hemiptera: Tingidae) relative to both control and severe-stress conditions [22], while drought altered herbivore performance and weakened indirect plant defenses in a sugar beet–aphid–parasitoid system [23]. These findings indicate that intermediate levels of plant stress may sometimes generate responses that cannot be predicted from a simple linear stress–fitness relationship. Particularly relevant to the present study, Maftoon et al. [24] recently demonstrated a nonlinear response of the parasitoid *Encarsia formosa* Gahan (Hymenoptera: Aphelinidae) to water availability in a tomato–*Trialeurodes vaporariorum*–parasitoid system. Moderate water stress corresponding to 70% of full irrigation enhanced parasitoid development, reproduction, and population performance, whereas severe stress at 50% of full irrigation reduced longevity and fecundity. Their study provides important evidence that irrigation management can influence biological control through plant-mediated bottom-up effects. However, a parasitoid and a predatory thrips occupy different ecological niches and interact with their herbivorous resources in different ways. Therefore, the response of predatory thrips to drought-induced changes in tomato and spider mites cannot be inferred directly from the response of *E. formosa*. Thus, experimental evaluation of the tomato–*T. urticae*–*S. longicornis* system is necessary.

The present study aimed to elucidate how plant water availability influences the life-history traits and demographic performance of the predatory thrips *S. longicornis* through plant-mediated bottom-up effects. Based on evidence that herbivores and their natural enemies can respond nonlinearly to changes in plant water status, we hypothesized that the demographic response of *S. longicornis* would depend on the severity of water stress rather than decline progressively with decreasing water availability. Specifically, we predicted that moderate water stress would maintain or enhance predator reproductive and demographic performance relative to well-watered control, whereas severe water stress would reduce adult longevity, fecundity, and reproductive output. By testing these predictions, we sought to clarify how variation in plant water availability influences the demographic potential of a natural enemy and contributes to our understanding of multitrophic interactions under water-limited conditions.

## 2. Results

Except for the eggs, the developmental duration of *S. longicornis* immature stages was not significantly affected by soil moisture treatment (Table 1). The total pre-adult developmental period ranged from 14.21 days under 70% FC to 15.56 days under 50% FC with a statistically significant treatment effect. Adult longevity was significantly reduced under severe water stress. Female longevity was similar under 100% FC (19.39 days) and 70% FC (20.14 days) but decreased significantly to 15.50 days under 50% FC; males showed the same pattern (Table 1).

These results were consistent with the fecundity data presented in Table 2. Total fecundity was significantly highest under 70% FC (62.27 offspring/female), followed by 100% FC (52.61 offspring/female), while the lowest fecundity occurred under 50% FC (41.33 offspring/female). Thus, moderate water stress resulted in greater lifetime reproductive output, whereas severe water stress substantially reduced reproduction. The oviposition period showed a similar response, lasting 15.68 days under 70% FC, significantly longer than 13.61 days under 100% FC and 10.38 days under 50% FC (Table 2). In contrast, APOP did not differ significantly among treatments. The proportion of female offspring was also statistically similar among soil moisture treatments, ranging from 0.55 to 0.60 (Table 2).

The results of population parameters analysis showed that the net reproductive rate (*R*_0_) was significantly affected by soil moisture treatment. The highest *R*_0_ was recorded under 70% FC (34.25 offspring/individual), followed by 100% FC (30.25 offspring/individual), while the lowest value was observed under 50% FC (24.80 offspring/individual). Significant differences in the finite rate of increase, intrinsic rate of increase and mean generation time were also found between the treatments (Table 3).

**Figure 1:**
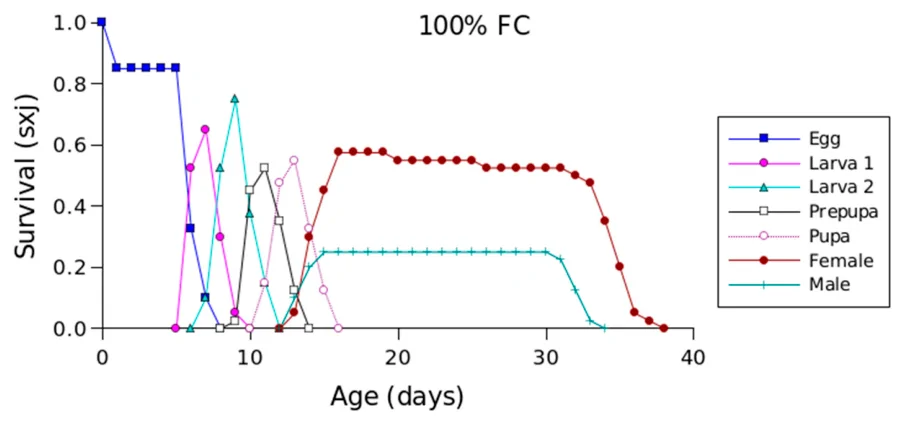

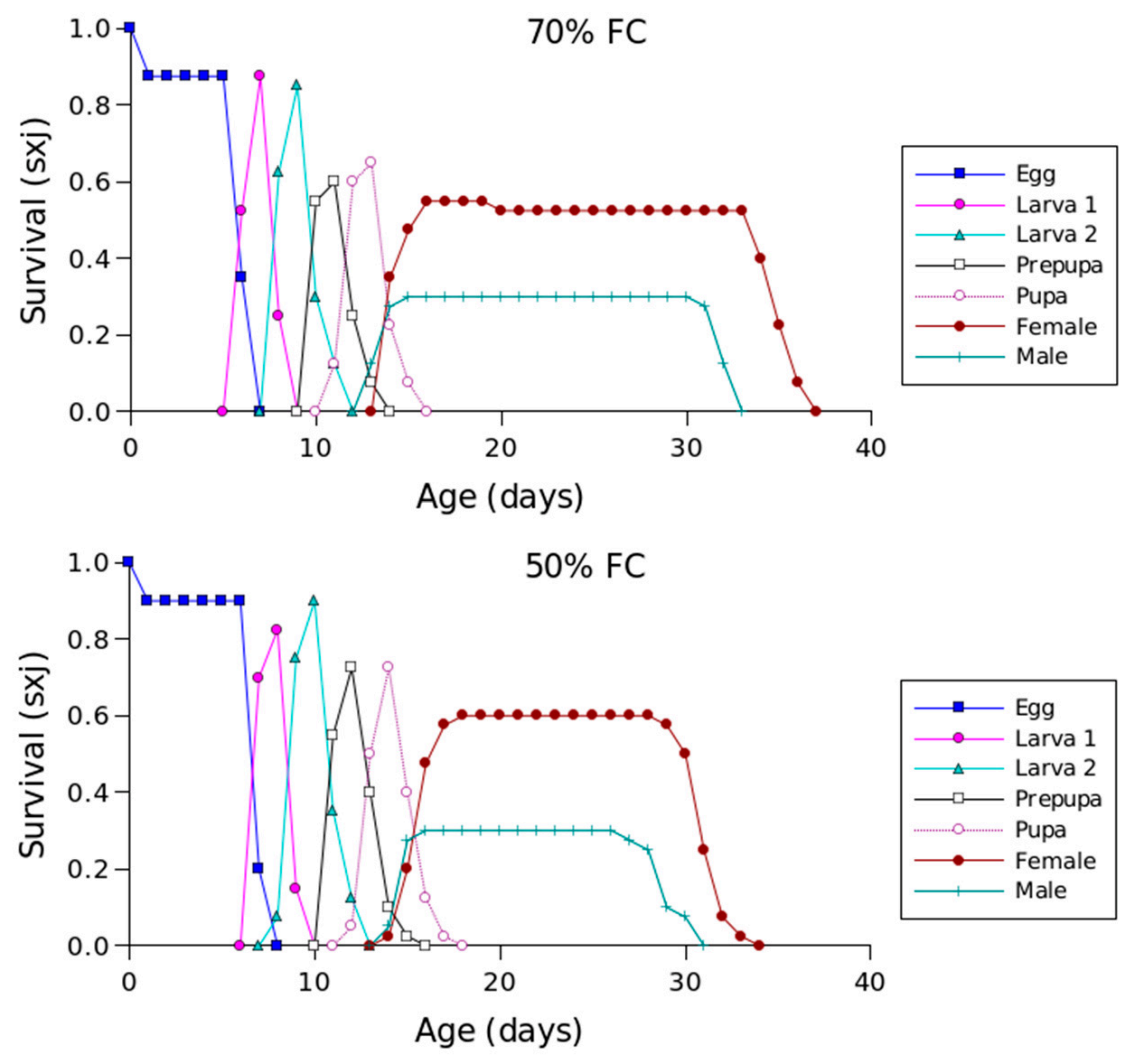
Age-stage specific survival rate (*s_xj_*) of *Scolothrips longicornis* on tomato plants under 100% FC, 70% FC, and 50% FC treatments. FC = field capacity.

We were able to accurately identify stage overlap and distinction due to differences in development rates in *S. longicornis* by using the *s_xj_*, which displays the probability that an individual will live to age *x* and stage *j* (Figure 1). At the onset of the adult stage, the probability that a newly laid egg survived to become an adult female under 50%, 70% and 100% FC was 0.60, 0.55 and 0.58, respectively, and the probability that it became an adult male was 0.30, 0.30 and 0.25, respectively. The last surviving females under 50%, 70% and 100% FC were observed at ages 34, 37 and 38 days, respectively, while the last surviving males were observed at ages 31, 33 and 34 days, respectively (Figure 1).

**Figure 2:**
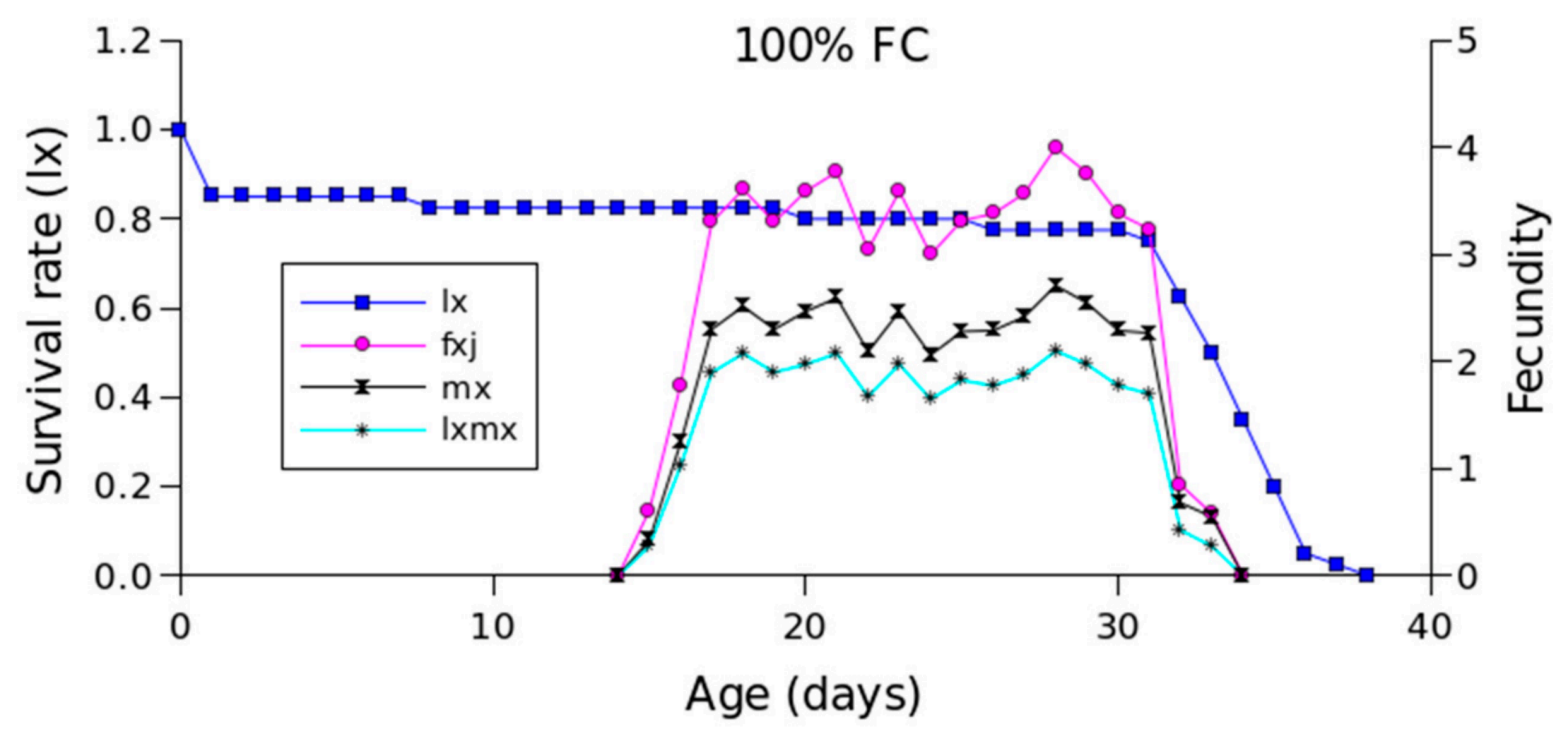

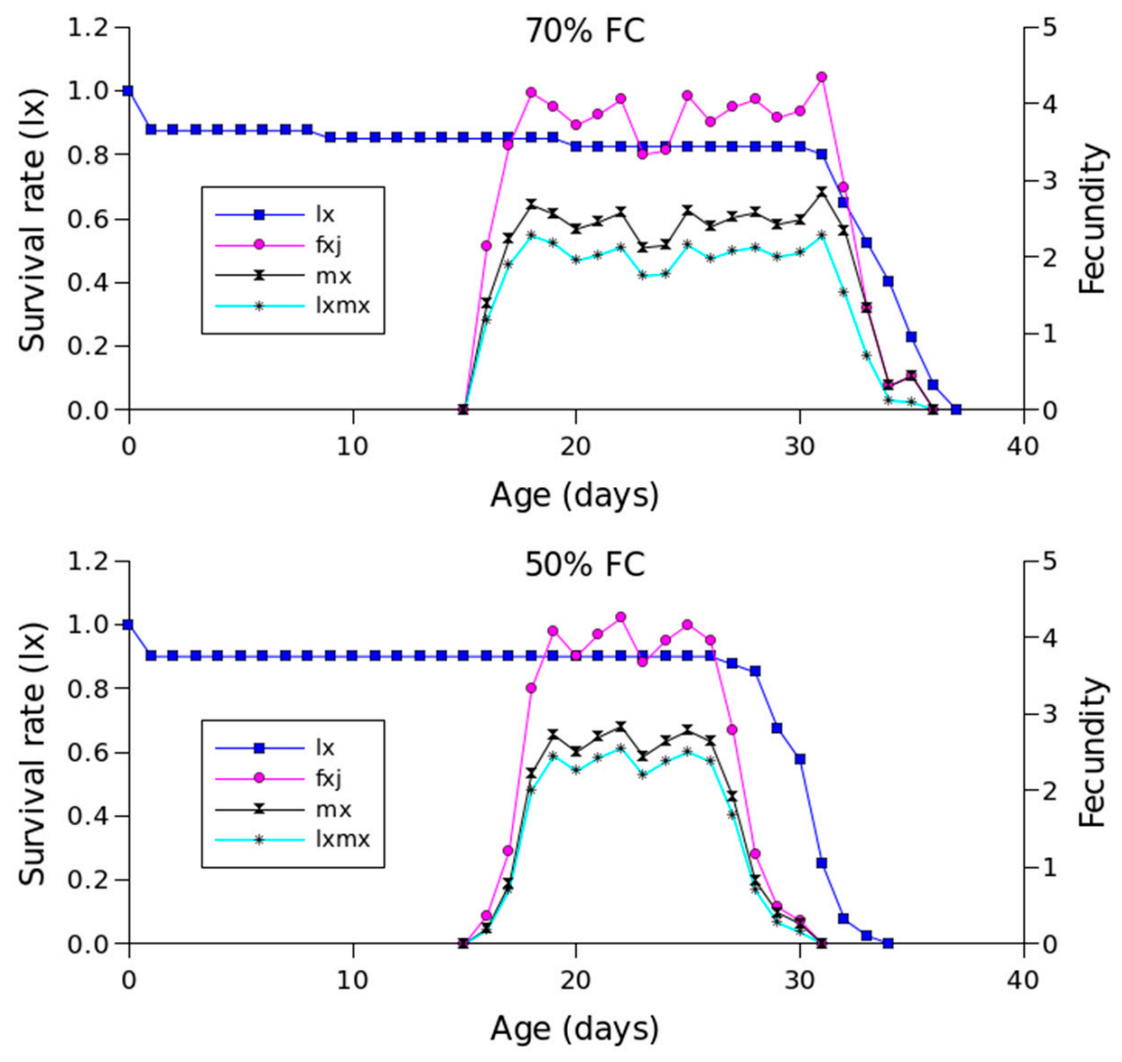
Age-specific survival rate (*l_x_*), age-specific fecundity (*m_x_*), age-specific maternity (*l_x_m_x_*), and age-stage-specific fecundity (*f_xj_*) of *Scolothrips longicornis* on tomato plants under 100% FC, 70% FC, and 50% FC treatments. FC = field capacity.

**Figure 3:**
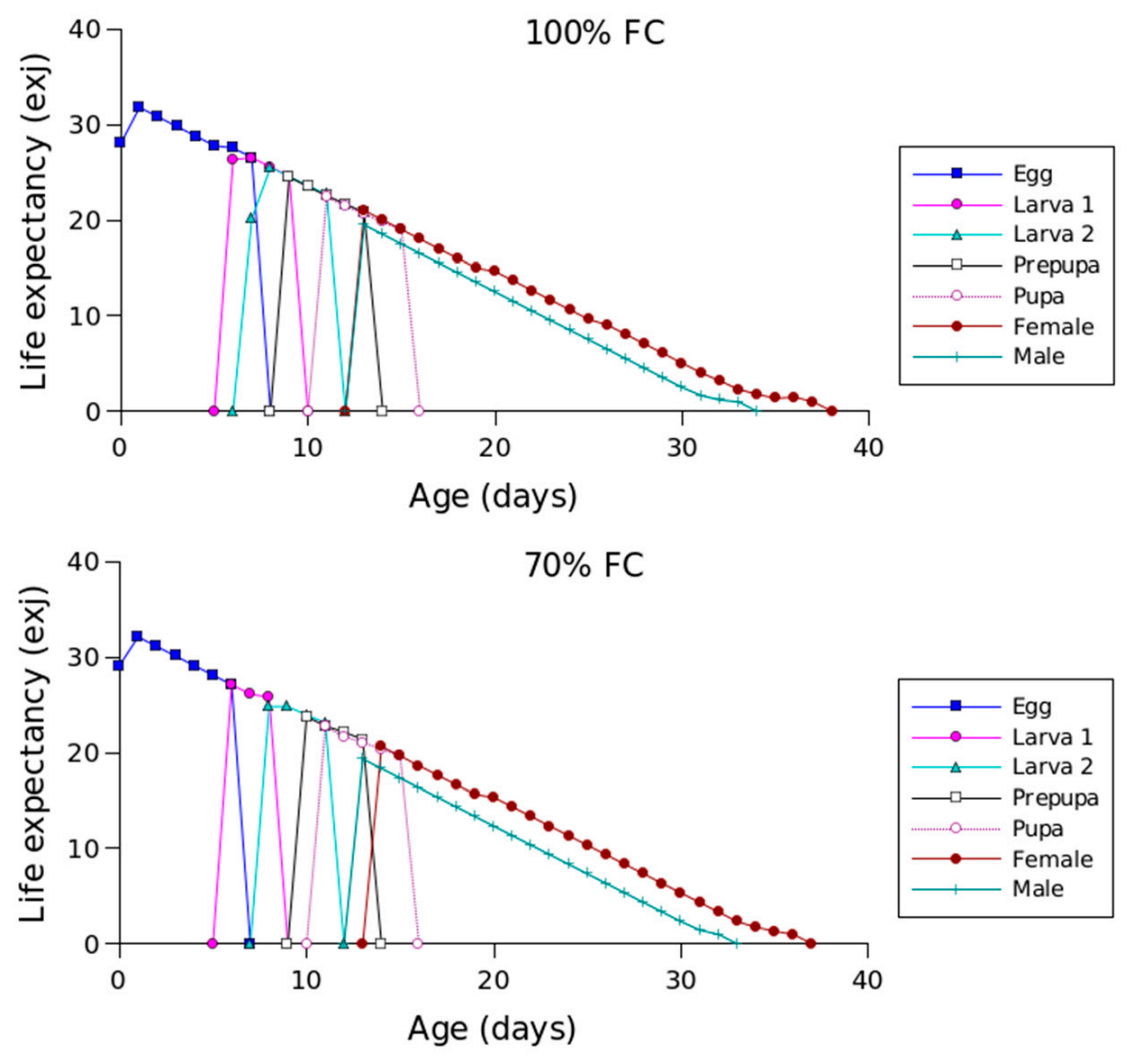

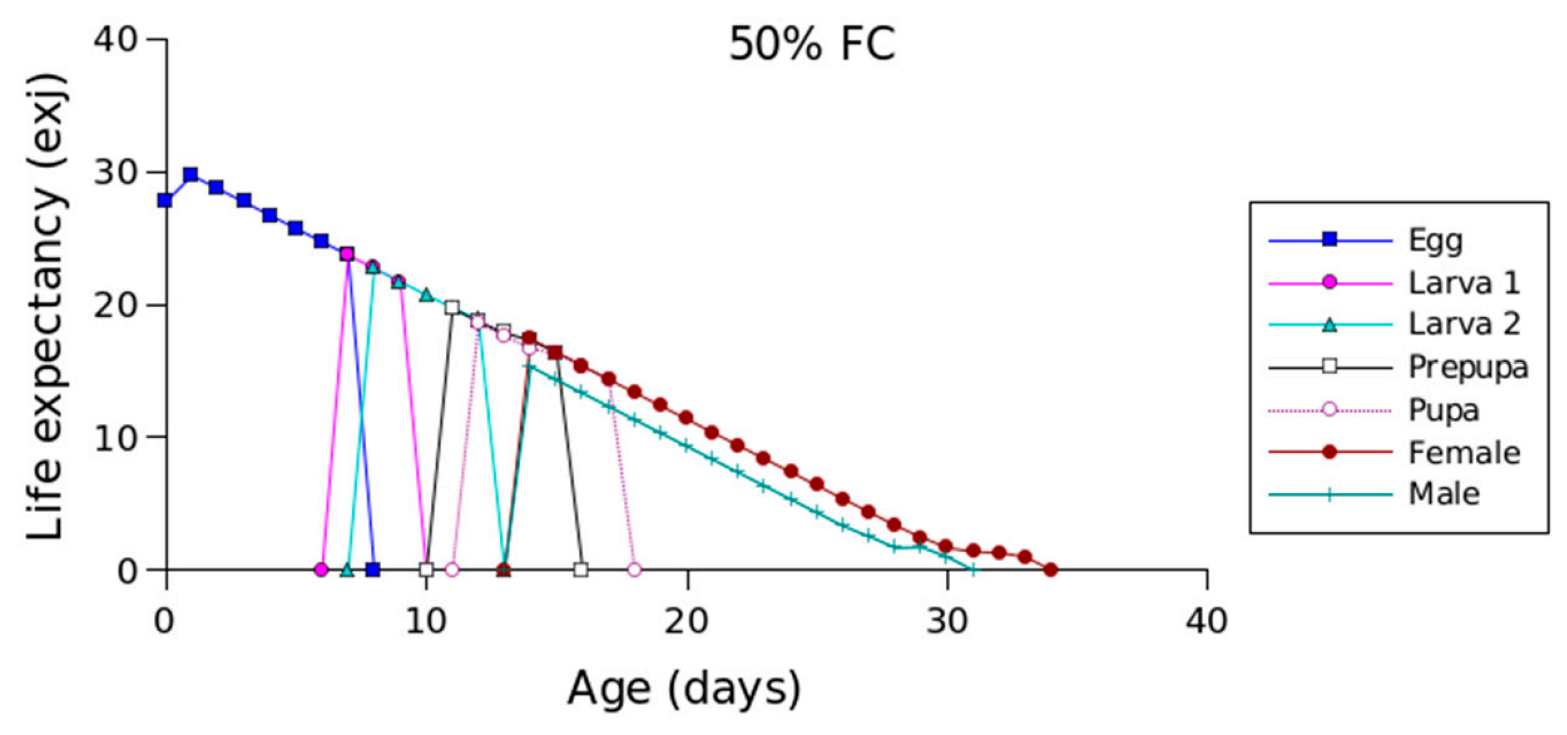
Age-stage-specific life expectancy (*e_xj_*) of *Scolothrips longicornis* on tomato plants under 100% FC, 70% FC, and 50% FC treatments. FC = field capacity.

**Figure 4:**
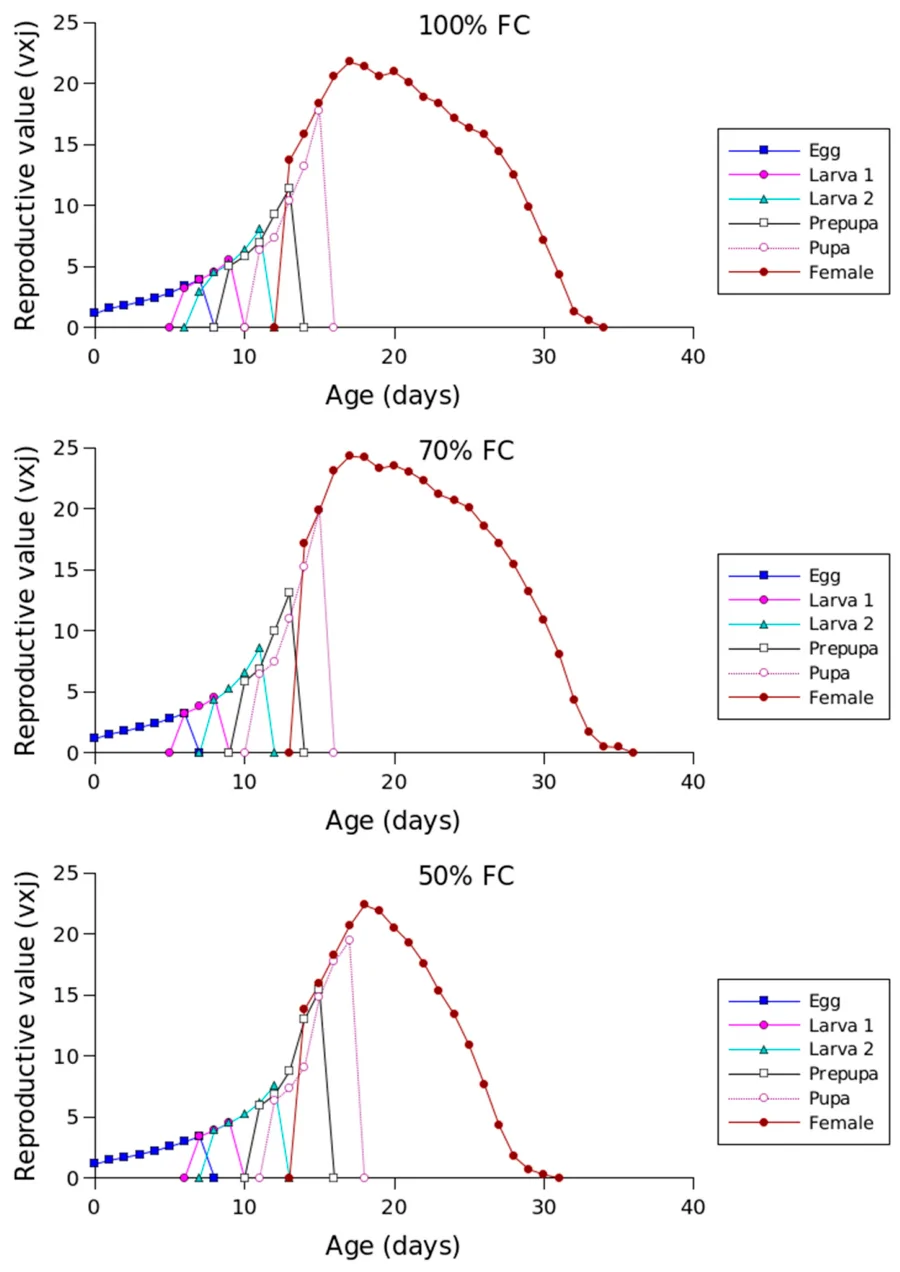
Age-stage-specific fecundity (*v_xj_*) of *Scolothrips longicornis* on tomato plants under 100% FC, 70% FC, and 50% FC treatments. FC = field capacity.

The *l_x_*, *m_x_*, *l_x_m_x_* and *f_xj_* values for the three treatments are displayed in Figure 2. Compared with all the treatments, 70% FC was associated with a longer oviposition period and a higher *m_x_* value. For 50% FC (day 23), 70% FC (day 32) and 100% FC (day 29), the highest female age-stage specific fecundity values were 2.83, 2.84, and 2.71 eggs, respectively. The age-stage-specific life expectancy (*e_xj_*) represents the expected remaining lifespan of an individual that has reached age *x* and stage *j*. For newly emerged adult females, the expected remaining lifespan was 15 days under 50% and 70% FC and 14 days under 100% FC (Figure 3). The maximum age-stage-specific fecundity (*v_xj_*), which represents the expected contribution of an individual at age x and stage j to future population growth by integrating its future survival and reproductive potential, was 22.36 at age 19 days under 50% FC, 24.30 at age 17 days under 70% FC and 21.77 at age 17 days under 100% FC (Figure 4).

**Figure 5:**
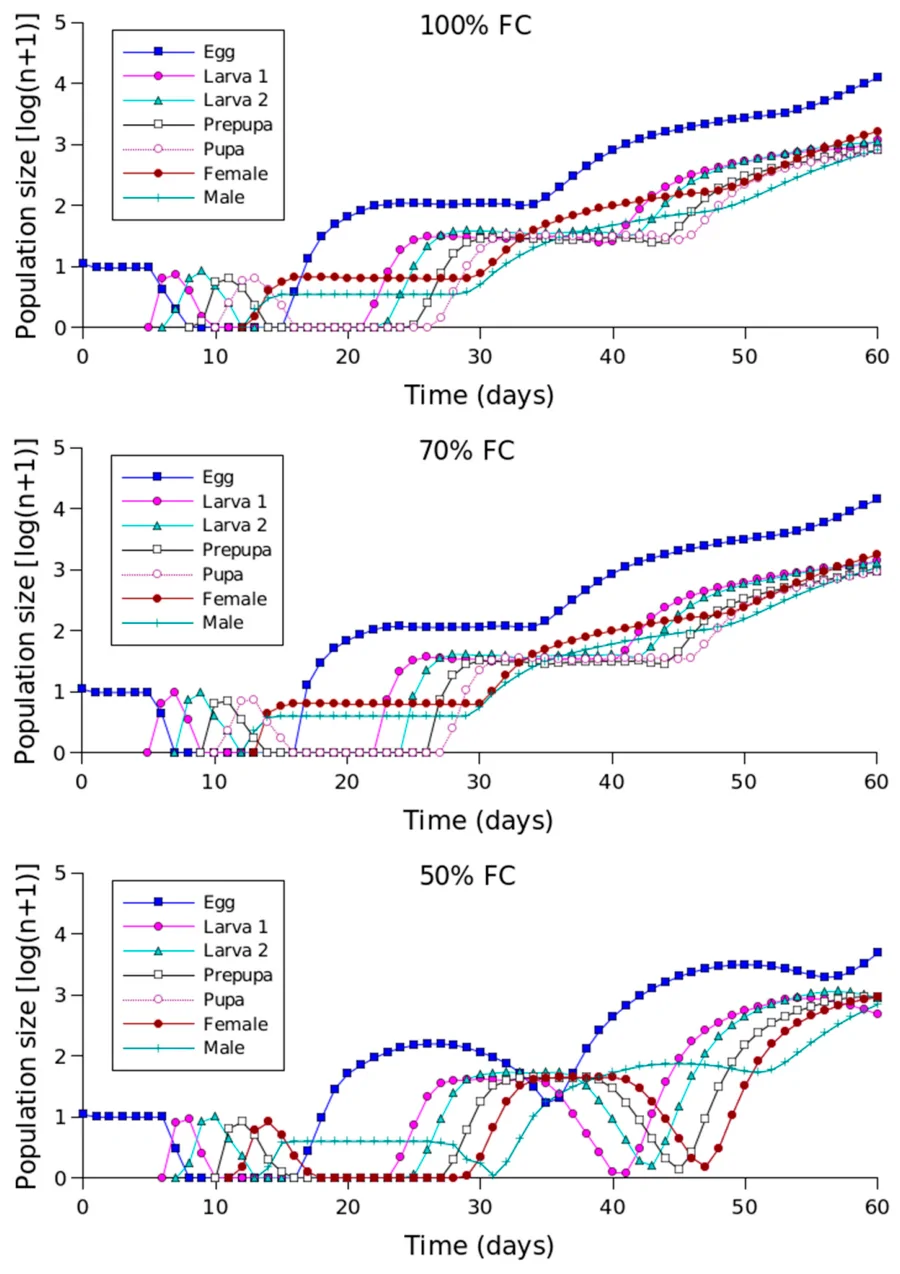
Changes in stage structure of *Scolothrips longicornis* on tomato plants under 100% FC, 70% FC, and 50% FC treatments. FC = field capacity.

**Figure 6:**
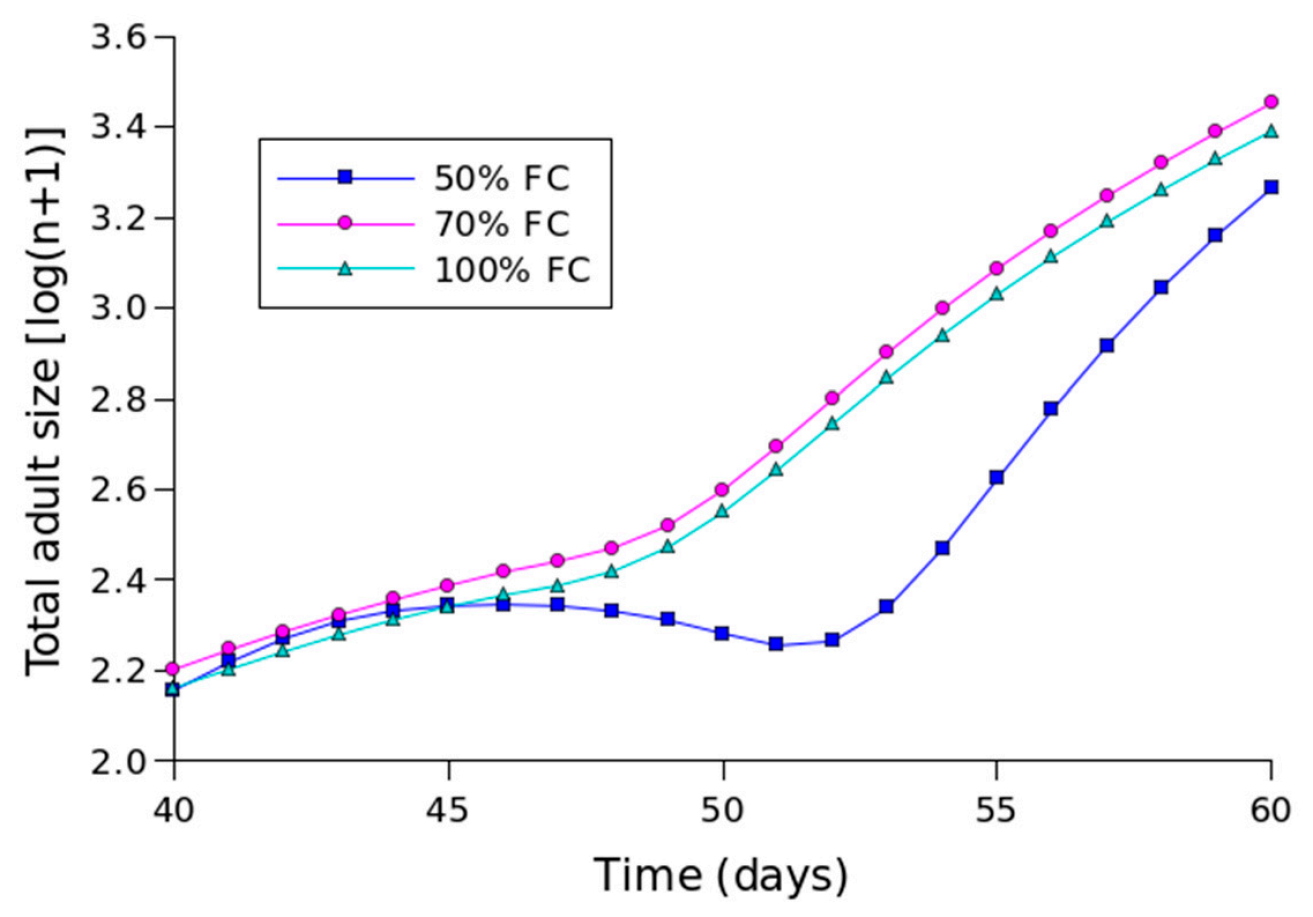
The total adult size of *Scolothrips longicornis* on tomato plants under 100% FC, 70% FC, and 50% FC treatments in 40–60-day time interval. FC = field capacity.

To simulate the changes in the population size and stage structure of *S. longicornis* fed on *T. urticae* eggs, data from life tables were utilized to project the population growth (Figure 5). It demonstrates that population growth is faster when predatory thrips were at 70% FC. The total predicted adult population size after 60 days was 3.265 in log scale (it approximated 1841 individuals) at 50% FC, 3.453 in log scale (it approximated 2838 individuals) at 70% FC, and 3.391 in log scale (it approximated 2461 individuals) at 100% FC (Figure 6).

## 3. Discussion

The present study demonstrated that plant water status can significantly influence the demographic performance of *S. longicornis* through plant-mediated effects, although the response was nonlinear. While immature development and survival were largely unaffected by soil moisture treatment, adult longevity, fecundity, and population parameters varied among treatments. Moderate water stress (70% FC) enhanced reproductive performance, whereas severe water stress (50% FC) reduced adult longevity and fecundity. These findings support the hypothesis that moderate levels of plant stress may improve the suitability of the prey-mediated environment for natural enemies, whereas more severe stress becomes detrimental. Such a pattern was, for example, observed in the tomato–white fly–*Encarsia formosa* system, where severe water stress significantly reduced adult parasitoid longevity, whereas moderate stress maintained or enhanced its fitness [24].

The absence of significant differences in immature developmental time and pre-adult survival suggests that juvenile stages of *S. longicornis* are relatively tolerant to variation in host-plant water status mediated through their prey. Similar stability of immature development under varying environmental conditions has been reported for *S. longicornis* [25] and other predatory arthropods [26], indicating that developmental processes may be less sensitive to indirect effects of plant stress than adult performance. In contrast, severe water stress significantly reduced adult longevity. Female longevity declined from approximately 20 days under control and moderate water stress to about 15 days under severe water stress, and males exhibited a similar response. A comparable pattern has been reported under heat stress, where adult longevity and reproductive traits were more strongly affected than immature development [19], suggesting that adult fitness components may represent a particularly sensitive stage in the response of predatory thrips to environmental stress.

The most pronounced treatment effects were observed in reproductive performance. Females maintained on plants subjected to moderate water stress exhibited a longer oviposition period and significantly higher fecundity than those maintained under either full irrigation or severe water stress. Consequently, the net reproductive rate was highest under 70% FC. Such responses are consistent with the view that moderate plant stress may alter plant physiology and herbivore quality in ways that benefit higher trophic levels [2]. The pattern observed here is broadly consistent with predictions derived from the Plant Stress Hypothesis and related conceptual frameworks, which propose that moderate stress can increase resource availability or nutritional quality for herbivores, whereas severe stress may reduce host suitability [2,10,27]. Because *S. longicornis* depends entirely on prey resources, any drought-induced changes in prey quality or availability are likely to cascade to predator performance.

Although reproductive output differed substantially among treatments, the differences in intrinsic rate of increase and finite rate of increase were less obvious. This reflects the fact that these demographic parameters integrate both reproduction and developmental timing. The relatively stable developmental rates observed across irrigation treatments likely reduced the magnitude of differences in population growth parameters despite substantial variation in fecundity and net reproductive rate. Nevertheless, population projection analyses indicated a tendency toward greater population growth under moderate water stress, suggesting that enhanced reproduction may ultimately translate into larger predator populations over time.

The mechanisms underlying these responses remain uncertain because neither tomato physiological traits nor spider mite population characteristics were quantified in the present study. Importantly, 100% FC should not necessarily be interpreted as the biologically optimal condition for tomato plants. Previous studies have shown that moderate water deficit can maintain or even improve certain aspects of tomato performance, including water-use efficiency, fruit quality, and resource allocation, whereas excessive or insufficient water availability may reduce plant vigor. Likewise, tomato responses to irrigation frequently exhibit nonlinear patterns, with intermediate moisture levels sometimes producing outcomes comparable to or better than those observed under full irrigation [28].

Water stress shifts plant hormone signaling, blocking jasmonic acid-mediated defenses while sometimes elevating salicylic acid or physical traits like callose deposition, which can alter plant feeder performance depending on stress intensity [29]. Response of *T. urticae* to drought-stressed host plants was found to be nonlinear, too [30,31]. It was also found that water stress induced tomato plant defense proteins while it also increased concentrations of essential amino acids and free sugars, improving the nutritional value of drought-stressed tomato plants for *T. urticae* and enhancing its population growth [12]. Therefore, the superior performance of *S. longicornis* under 70% FC may have resulted from changes in plant physiology, prey quality, prey abundance, or a combination of these factors. Without direct measurements of plant and prey responses, these mechanisms remain speculative.

From an applied perspective, the results indicate that moderate water limitation does not necessarily impair the demographic performance of this important predator and may even enhance its reproductive potential. In regions where water-saving irrigation strategies are increasingly adopted because of water scarcity and climate change, understanding such multitrophic consequences is essential for integrating biological control into sustainable crop management programs.

## 4. Materials and Methods

### 4.1. Host Plant Cultivation

The experiments were conducted using tomato plants of the cultivar SUN 6216 F1, which was selected because of its reported tolerance to drought conditions. Tomato seeds were initially sown in seedling trays containing a sterilized cocopeat–perlite mixture at a ratio of 3:1 (*v*/*v*). Seedlings were maintained in a controlled-environment growth chamber at 25 ± 1 °C, 65 ± 5% relative humidity, and a 16:8 h L:D photoperiod. The photoperiod was provided by LED lamps (Pars Shahab, Tehran, Iran). At 17 days after sowing (DAS), uniformly developed seedlings at the 4–5 true leaf stage were transplanted individually into 3.5 L plastic pots containing the same cocopeat–perlite substrate (3:1, *v*/*v*). Plants were supplied with full irrigation for six days after transplanting to allow adequate establishment and root development. Subsequently, plants were randomly allocated to one of three soil moisture treatments: 100% field capacity (FC; well-watered control), 70% FC (moderate water stress FC), and 50% FC (severe water stress FC). Field capacity (FC), defined as the maximum amount of water the substrate can hold after excess water has drained away [32], was determined gravimetrically. Briefly, the substrate was saturated with water and allowed to drain for 24 h, after which the corresponding pot weight was measured by digital scales (EK-610i, A&D Company Ltd., Tokyo, Japan) and recorded as the reference weight for 100% FC. The target pot weights corresponding to the 70% and 50% FC treatments were subsequently calculated from this reference weight. Throughout the experiment, each pot was weighed daily, and water loss caused by evapotranspiration was compensated by adding the required amount of water to restore the respective target moisture level. Each soil moisture treatment consisted of 40 independently maintained plants (n = 40), arranged in a completely randomized design.

### 4.2. Rearing of Prey and Predator

A laboratory colony of *T. urticae* was established from individuals collected from infested tomato plants in a commercial greenhouse in Varamin, Tehran Province, Iran. The mites were subsequently reared continuously on tomato plants of the same cultivar (Sun 6216 F1) in nylon 100-mesh-covered cages under controlled environmental conditions.

To obtain synchronized cohorts of *T. urticae* eggs, adult mites were allowed to oviposit on healthy tomato leaves for 24 h. Individual leaves were enclosed using customized cages made from plastic Petri dishes (diameter 10 cm, height 1.5 cm). A circular opening (diameter 5 cm) was made in each cage lid and covered with fine nylon 100-mesh to provide ventilation while preventing the escape of mites. A lateral slit in both the lid and base of the cage was made, allowing passage of the leaf petiole. The space between the opening and the petiole was sealed with dry cotton wool. After the 24 h oviposition period, adult mites were removed, and the leaves containing eggs were isolated. This procedure was repeated for five successive generations to reduce potential variation associated with parental generation and rearing history. The fifth-generation synchronized colony was used as the prey source for the experiments.

Adults of *S. longicornis* were collected from tomato plants in the same commercial greenhouse in Varamin. The predators were transferred to mesh-covered rearing cages containing potted tomato plants infested with *T. urticae*, which served as their prey. The *S. longicornis* colony was maintained on the tomato–*T. urticae* system for approximately 10 generations before the experiments were initiated. The prey and predator colonies, as well as the experimental insects, were maintained at 25 ± 1 °C, 65 ± 5% relative humidity, and a 16:8 h L:D photoperiod.

### 4.3. Life-Table Experiment

To evaluate the plant-mediated effects of water availability on predator performance, a continuous tritrophic system consisting of tomato plants, *T. urticae*, and *S. longicornis* was established under each soil moisture treatment. Tomato plants assigned to the 100% FC, 70% FC, and 50% FC treatments were maintained under their respective irrigation conditions throughout the experiment.

When the plants had developed 8–10 leaves, two leaves per plant were enclosed using the previously described Petri-dish cages. Ten adult female *T. urticae* were introduced into each cage and allowed to oviposit for 24 h. After the females were removed, the synchronized prey eggs remained on the leaves and served as the food source for the predator.

Newly emerged (<24 h old) male and female *S. longicornis* adults (10–15 individuals per cage) were then introduced into the same cages and allowed to oviposit for 24 h, after which all adult predators were removed. The tomato plants bearing both *T. urticae* eggs and newly deposited *S. longicornis* eggs were subsequently maintained under controlled environmental conditions (25 ± 1 °C, 65 ± 5% RH, and a 16:8 h L:D photoperiod).

Forty *S. longicornis* eggs were randomly selected from each soil moisture treatment and kept individually in separate leaf cages distributed across 20 plants. The other 20 plants of the same treatment served for preparation of prey eggs.

Each individual was monitored daily from egg hatch until death. Developmental stage, survival, adult longevity, and fecundity were recorded at 24 h intervals. Throughout the experiment, predators were provided with *T. urticae* eggs originating from the corresponding soil moisture treatment. Because the leaves initially contained high densities of spider mite eggs, prey availability was generally sufficient; however, leaf cages were inspected daily, and additional *T. urticae* eggs originating from plants maintained under the same soil moisture treatment were added whenever necessary to maintain a minimum density of approximately 100 prey eggs per leaf cage. The predator cohort was therefore generated and maintained within the same soil moisture-treated tomato–spider mite system, preserving the plant-mediated component of the experiment throughout the life-table observations.

### 4.4. Statistical Analysis

The data were analyzed using the age-stage, two-sex life table theory using the computer program TWOSEX MS-Chart, Version 2024.05.07 [33,34]. A bootstrap sampling technique with 100,000 iterations was used to estimate the standard errors and variances of biological traits and population parameters. Differences between treatments were evaluated using the paired bootstrap test implemented in TWOSEX MS-Chart, in which the same bootstrap resample was generated for both treatments to preserve the paired comparison. Statistical significance was determined from the bootstrap distribution of the paired differences, with differences considered significant when the 95% confidence interval did not include zero (equivalent to *p* < 0.05). In addition, the life table data from all treatments were used to project the population growth and the structure of each stage of *S. longicornis* over a period of 60 days with an initial population of 10 eggs. The simulation was done using the program TIMING-MSChart, Version 2025.02.18 [35] based on the method explained in [36]. Table A1 presents the list of all estimated demographic parameters together with their definitions and formulae.

## 5. Conclusions

This study demonstrates that plant-mediated effects of water availability on *S. longicornis* are nonlinear. Moderate water stress (70% FC) enhanced fecundity, prolonged oviposition, and increased the net reproductive rate of the predator, whereas severe water stress (50% FC) reduced adult longevity and reproductive performance. In contrast, immature survival remained largely unaffected by soil moisture regime.

These findings suggest that moderate water limitation may be compatible with, or even favorable for, the demographic performance of *S. longicornis* in tomato production systems, whereas severe water stress may compromise predator fitness. The results highlight the importance of considering plant water status when evaluating the potential effectiveness of natural enemies in biological control programs under water-limited conditions.

Future research should combine measurements of plant physiological responses, prey performance, and predator demography to identify the mechanisms responsible for the enhanced reproductive performance observed under moderate water stress. In addition, experiments conducted under greenhouse and field conditions should determine whether the demographic advantages observed here translate into improved suppression of *T. urticae* populations and greater biological control efficacy.

## Figures and Tables

**Table 1:** Mean (± SE) developmental time, survival rate, and adult longevity of *Scolothrips longicornis* on tomato plants under 100% FC, 70% FC, and 50% FC treatments. FC = field capacity.

Stage	100% FC	70% FC	50% FC
Egg (days)	6.61 ± 0.16 b ^1^	6.50 ± 0.11 b	7.33 ± 0.1 a
Larva I (days)	1.78 ± 0.09 a	1.91 ± 0.06 a	1.79 ± 0.08 a
Larva II (days)	2.39 ± 0.10 a	2.27 ± 0.10 a	2.67 ± 0.10 a
Prepupa (days)	1.87 ± 0.07 a	1.86 ± 0.07 a	2.00 ± 0.09 a
Pupa (days)	1.96 ± 0.04 a	1.95 ± 0.05 a	2.08 ± 0.08 a
Pre-adult (days)	14.61 ± 0.20 b	14.21 ± 0.14 b	15.56 ± 0.15 a
Pre-adult survival (probability)	0.83 ± 0.06 a	0.85 ± 0.06 a	0.90 ± 0.05 a
Female longevity (days)	19.39 ± 0.79 a	20.14 ± 0.70 a	15.50 ± 0.10 b
Male longevity (days)	18.70 ± 0.15 a	18.67 ± 0.19 a	14.42 ± 0.26 b

^1^ Values followed by different letters within the same row are significantly different (paired bootstrap test, *p* < 0.05).

**Table 2:** Mean (±SE) lifespan, fecundity, total preoviposition period (TPOP), adult preoviposition period (APOP), and oviposition period of *Scolothrips longicornis* on tomato plants under 100% FC, 70% FC, and 50% FC treatments. FC = field capacity.

Biological Parameter	100% FC	70% FC	50% FC
Female lifespan (days)	34 ± 0.80 a ^1^	34.64 ± 0.73 a	31.38 ± 0.22 b
Male lifespan (days)	32.50 ± 0.27 a	32.33 ± 0.19 a	29.33 ± 0.36 b
Fecundity (eggs)	52.61 ± 2.36 b	62.27 ± 2.60 a	41.33 ± 0.46 c
APOP (days)	1.96 ± 0.10 a	2.05 ± 0.08 a	1.96 ± 0.04 a
TPOP (days)	16.57 ± 0.20 a	16.55 ± 0.14 a	17.83 ± 0.20 b
Oviposition period (days)	13.61 ± 0.55 b	15.68 ± 0.58 a	10.38 ± 0.15 c
Sex ratio (proportion of females)	0.58 ± 0.08 a	0.55 ± 0.08 a	0.60 ± 0.08 a

^1^ Values followed by different letters within the same row are significantly different (paired bootstrap test, *p* < 0.05).

**Table 3:** Mean (±SE) of population parameters of *Scolothrips longicornis* on tomato plants under 100% FC, 70% FC, and 50% FC treatments. FC = field capacity.

Population Parameter	100% FC	70% FC	50% FC
*r* (day^−1^)	0.147 ± 0.007 b ^1^	0.149 ± 0.006 a	0.141 ± 0.006 c
*λ* (day^−1^)	1.158 ± 0.008 b	1.161 ± 0.007 a	1.151 ± 0.007 c
*R*_0_ (offspring/individual)	30.25 ± 4.33 b	34.25 ± 5.11 a	24.80 ± 3.21 c
*T* (days)	23.21 ± 0.22 b	23.65 ± 0.16 a	22.84 ± 0.20 c

^1^ Values followed by different letters within the same row are significantly different (paired bootstrap test, *p* < 0.05).

## Data Availability

The data that support the findings of this study are available upon request from the corresponding author.

## Abbreviations

The following abbreviations are used in this manuscript:

APOP	Adult pre-oviposition period
TPOP	Total pre-oviposition period
L:D	Light–dark
RH	Relative humidity
FC	Field capacity

## Appendix A

**Table A1:** Population parameters, their definitions, and equations used in their calculations.

Parameter and Equation	Definition
Adult pre-ovipositional period (APOP)	The period between adult emergence and first oviposition.
Total pre-ovipositional period (TPOP)	The period from birth to first oviposition.
Age stage-specific survival rate (*s_xj_*) $s_{\mathrm{xj}}=\frac{n_{\mathrm{xj}}}{n_{01}}$	The probability that a newly laid egg will survive to age *x* and stage *j*. *n_xj_* is the number of individuals that survive to age *x* and stage *j*, and *n*_01_ is the number of newborn offspring used at the beginning of the life table study [37].
Age stage-specific fecundity (*f_xj_*)	The mean number of offspring produced by individuals at age *x* and stage *j* [33].
Age-specific fecundity (*m_x_*) $m_{x}=\left(\sum_{j=1}^{m}s_{\mathrm{xj}}f_{\mathrm{xj}}\right)/\left(\sum_{j=1}^{m}s_{\mathrm{xj}}\right)$	The mean number of offspring produced by individuals at age *x* [33].
Net reproductive rate (*R*_0_) $R_{0}=\sum_{x=0}^{\infty }\sum_{j=1}^{m}s_{\mathrm{xj}}f_{\mathrm{xj}}=\sum_{x=0}^{\infty }l_{x}m_{x}$	The total number of offspring that an average individual (including females, males, and those that died in the immature stage) can produce during its lifetime [38].
The intrinsic rate of increase (*r*) $\sum_{x=0}^{\infty }e^{-\mathrm{rx}}l_{x}m_{x}=1$	The population growth rate as time approaches infinity and the population reaches a stable age-stage distribution (SASD). The population size will increase at the rate of *e^r^* per time unit. It is calculated using the Euler–Lotka equation with age indexed from 0 [38].
The finite rate of increase (*λ*) *λ* = *e^r^*	The population growth rate as time approaches infinity and the population reaches a stable age-stage distribution. The population size will increase at the rate of *λ* per time unit [38].
Mean generation time (*T*) $T=\frac{\ln R_{0}}{r}=\frac{\ln R_{0}}{\ln\lambda }$	The period that a population requires to increase to *R*_0_-fold of its size as the time approaches infinity and the population settles down to a stable age-stage distribution [38].
Age stage-specific life expectancy (*e_xj_*) $e_{\mathrm{xj}}=\sum_{i=x}^{\infty }\sum_{y=j}^{k}{\mathrm{s’}}_{\mathrm{iy}}$	The duration for which an individual of age *x* and stage *j* is expected to survive. *s′_iy_* is the probability that an individual of age *x* and stage *j* will survive to age *i* and stage *y* and it is calculated by assuming *s_xj_* = 1 [39].
Age stage-specific reproductive value (*v_xj_*) $v_{\mathrm{xj}}=\frac{e^{r(x+1)}}{s_{\mathrm{xj}}}\sum_{i=x}^{\infty }e^{-r(i+1)}\sum_{y=j}^{k}{s\prime }_{\mathrm{iy}}f_{\mathrm{iy}}$	The reproductive value is described as the contribution of an individual of age *x* and stage *j* to the future population. For immature stages such as the larval stage, the reproductive value is not an actual reproductive output, but rather a projection of their expected future reproductive contribution if they survive to adulthood [40].
